# Developing a framework for gathering and using service user experiences to improve integrated health and social care: the SUFFICE framework

**DOI:** 10.1186/s13104-016-2230-0

**Published:** 2016-09-08

**Authors:** Vicky Ward, Lisa Pinkney, Gary Fry

**Affiliations:** Leeds Institute of Health Sciences, University of Leeds, 101 Clarendon Road, Leeds, LS2 9LJ UK

**Keywords:** Integrated care, Service user experience, Health care, Social care, Service improvement

## Abstract

**Background:**

More people than ever receive care and support from health and social care services. Initiatives to integrate the work of health and social care staff have increased rapidly across the UK but relatively little has been done to chart and improve their impact on service users. Our aim was to develop a framework for gathering and using service user feedback to improve integrated health and social care in one locality in the North of England.

**Methods:**

We used published literature and interviews with health and social care managers to determine the expected service user experiences of local community-based integrated teams and the ways in which team members were expected to work together. We used the results to devise qualitative data collection and analysis tools for gathering and analyzing service user feedback. We used developmental evaluation and service improvement methodologies to devise a procedure for developing service improvement plans.

**Findings:**

We identified six expected service user experiences of integrated care and 15 activities that health and social care teams were expected to undertake. We used these to develop logic models and tools for collecting and analysing service user experiences. These include a narrative interview schedule, a plan for analyzing data, and a method for synthesizing the results into a composite ‘story’. We devised a structured service improvement procedure which involves teams of health and social care staff listening to a composite service user story, identifying how their actions as a team may have contributed to the story and developing a service improvement plan.

**Conclusions:**

This framework aims to put service user experiences at the heart of efforts to improve integration. It has been developed in collaboration with National Health Service (NHS) and Social Care managers. We expect it to be useful for evaluating and improving integrated care initiatives elsewhere.

**Electronic supplementary material:**

The online version of this article (doi:10.1186/s13104-016-2230-0) contains supplementary material, which is available to authorized users.

## Background

In the United Kingdom (UK) a rising population of older adults and a significant increase in the number of people with complex health needs means that more people than ever are receiving care and support from both health and social care services. Recent figures show that 15 million people have one or more long-term conditions and their care accounts for 70 % of the primary and acute care budget in England [1] whilst around 1.1 million people receive care at home, 80 % of whom are state supported [2]. The coordination of care and support for these people has historically been poor resulting in people falling through gaps in service provision, being ‘bounced around’ different services and having to explain themselves and their needs multiple times [3]. Poor coordination also results in duplication between health and social care services and unnecessary hospital stays, further increasing the financial burden on the system [4]. As a result there is currently a significant policy drive towards integrating health and social care support [5] and improving the experiences of service users with complex needs [6].

A range of integrated care initiatives have been implemented across the UK in recent years. In 2009 the Department of Health launched a 2-year pilot programme to explore and evaluate different models of integrated care [7]. In 2013 this was followed by the naming of 14 ‘integration pioneers’ across the UK where innovative ways of delivering coordinated care and bringing services closer together are being pursued [8]. These and other sites have used a range of approaches including multidisciplinary meetings [9], risk profiling and case management [10] and pooled service and commissioning budgets [11]. The majority of initiatives in the UK have tended to take place at a micro level where providers seek to deliver integrated care for individual service users through care co-ordination, care planning, use of technology and other approaches [12]. Such initiatives include setting up community-based co-located teams of nurses, social workers, occupational therapists and physiotherapists [13–15].

In an effort to learn lessons about how best to provide integrated health and social care and the impact of integrated working, recent initiatives have been the subject of a number of evaluations. Evaluative activities have largely focused on emergency hospital admissions and the barriers to integrated working from the perspective of health and social care staff [10, 16, 17] and findings to date have not been especially positive. The national evaluation of the UK’s integration pilot programme, for instance, showed no evidence that pilot sites were reducing the level of emergency hospital care [7], whilst a number of studies have highlighted a range of barriers to integrated working including professional/disciplinary mismatches, lack of clarity about the purpose of integration, lack of understanding and clarity about each other’s roles and the use of rhetorical claims and ideals to quash real experiences [14].

There has been less emphasis on how integrated ways of working are impacting on service users and how they can be designed to provide service users with a better experience of care. Evidence to date suggests that there is frequently a mismatch between the aims and impact of integration on service user experiences [7, 18], but there is general agreement that service user experiences of integrated care are not well defined or appropriately captured, especially by those organisations who are actively developing and implementing integrated care initiatives [3, 19]. Indeed, previous evaluative efforts have been criticized by service users and other stakeholders for focusing on descriptions of what integrated care should look like and what organisations should do, rather than on the voices and experiences of service users [19].

By failing to capture how service users are experiencing efforts to improve integration and coordination, organisations are also missing a potentially powerful catalyst for improving integrated working. During recent consultations, service users and other stakeholders across the UK were clear that efforts to evaluate service user experiences of integrated care should be used for driving improvement and encouraging better communication and joint working between health and social care professionals [19]. This is supported by evidence on interprofessional teamworking, which shows that having a shared sense of purpose focused around improving outcomes and experiences for service users is a key enabler of interprofessional teamwork [20, 21]. As Cameron et al. [22] found, expressing the benefits of joint working in terms of client outcomes/experiences helps staff to recognise that they need to work together to achieve these.

This paper focuses on the development of a framework for gathering and using service user feedback to improve integrated working between health and social care staff. Named SUFFICE (Service User Feedback Framework for Improving integrated CarE), the framework was developed at the request of health and social care organisations in a city in the North of England, who recognized their need to understand how service users were experiencing their local efforts to improve coordination and integrated working across the city and respond to those experiences. In the remainder of this paper we detail the methods used to develop the framework and describe the contents of the framework.

## Methods

### Developing the framework

#### Setting

In early 2012 the local vision for integration in our study site proposed that General Practitioners (GPs), health workers and social care staff would work side-by-side in close knit teams, identifying levels of risk, sharing information and taking a joint approach to supporting older people and those with long-term conditions. One of the first strands of work was the co-location of health and social care staff into 12 ‘integrated neighbourhood teams’, each of which served a specific geographical area. Teams were set up over a 12 month period between February 2012 and February 2013. These teams were chosen as the focus for our framework because they were seen as a particularly important element of the local integration effort, were a relatively well-defined intervention which could be distinguished from the usual ways of working, and offered a forum to engage staff in improving integrated working by reflecting on service user feedback. We were asked to design the framework in early 2013.

#### Design

The overall aim of our work was to design a practical and user-friendly framework which could be used by those commissioning and delivering integrated health and social care services to understand and respond better to the experiences of service users and their carers. The expectation of those who were setting up the teams was that they would develop and evolve over time. This led us to select developmental evaluation approaches as the basis for our work since these aim to support the development and adaptation of interventions in dynamic environments by focusing on the co-creation of useful and practical evaluation tools [23]. Our approach meant that we worked closely with staff from relevant health, social care and service user organisations throughout the project. This included establishing a project management group which included representatives from the research team and from local health, social care and voluntary sector organisations. This provided a forum for reporting progress back to the organisations who had requested the framework, and ensured that the framework itself would be fit-for-purpose.

##### Phase 1: understanding and uncovering expectations about integrated care experiences and practices

We divided the project into several phases. In the first phase we focused on understanding service user experiences of and expectations about integrated care and what effective interprofessional teamworking looks like. We operationalised these as two inter-related questions to help us better communicate our focus to our local partners: ‘What experiences can we expect integrated working to deliver for service users?’ and ‘How should we expect integrated neighbourhood teams to work together?’

In line with our developmental evaluation approach, our overall aims in this phase of the work were to determine the type of service user feedback that would be useful for developing integrated working, the type of activities that integrated teams could be encouraged to engage in, and develop a shared understanding (with our local partners) of how integrated neighbourhood teams could improve service user experiences of care.

We undertook three activities to enable us to answer these questions. First, discussions with our local partners led us to identify two initiatives (one local and one national) to develop outcome frameworks for integrated care. The local framework was based on interviews with service users and staff about what they wanted and expected from local integration efforts [24]. The national initiative was undertaken by National Voices (a coalition of health and social care charities in England) and involved interviewing and working with service users to produce narratives of person-centred, coordinated care [25]. We used both of these sources to develop initial lists of service user experiences and teamworking attributes and practices which could be associated with integrated care.

Second, we conducted two scoping reviews. These were specifically requested by our local partners in order to help them understand the current knowledge base on integrated working. Our first review focused on published accounts of service user’s experiences of integrated care. Our second review focused on published accounts of how staff from different organisational and professional backgrounds work together and the markers of effective teamworking. Our search strategies and results can be seen in Table 1.

**Table 1:** Scoping review search criteria and results

	Service user experiences	Interprofessional teamworking
Eligibility criteria	Published papers describing service user experiences of integration in health and social care 2008–2013 English language	Published papers describing integrated teamworking between health and/or social care professionals in community settings 2003–2013 English language
Databases	MEDLINE EMBASE HMIC Social care online ASSIA	MEDLINE EMBASE HMIC PSYCInfo ASSIA Social services abstracts PAIS international Scopus (health sciences, social science)
Hand searches	International journal of integrated care Journal of integrated care	International journal of integrated care Journal of integrated care
Search terms	Social care AND health AND integrated	Health OR social care AND [integrat* AND team* AND multi*] AND [community OR primary care] AND [case study OR evaluat* OR interview OR ethnograph* OR focus group OR survey OR questionnaire OR observat*]
Inclusion/exclusion criteria	Include: papers which provide empirical evidence of service user experiences Exclude: papers which only focus on staff perceptions of service user experiences, service or system-level outcomes, descriptions of integration schemes	Include: papers which provide empirical evidence of how multiprofessional teams work together to provide care to service users in community settings Exclude: theoretical papers and papers which focus on single professional groups
No. of returned papers	413	904
No. of papers meeting inclusion criteria	47	63

Two team members (VW and LP) read the papers which met our inclusion criteria in full and worked together to extract and summarise service user’s experiences of integrated care and the features of integrated teamworking and develop these into two sets of short statements. We also used the results of the reviews to produce short documents for our project partners which summarised the knowledge base in relation to these aspects of integrated care.

Our third activity involved conducting interviews with 15 local commissioning and service provider managers, including members of the local Integrated Health and Social Care Board. We decided not to interview service users since we were confident that the work undertaken locally and nationally adequately captured service user expectations about integrated care and we did not wish to overburden local service users. Written consent for the interviews was given by all participants. We asked interviewees what they hoped that service users would say after receiving care from an Integrated Neighbourhood Team and what the teams would need to do to achieve those positive experiences. Interviews were recorded and transcribed in full. Two team members (VW and LP) categorized interview material into ‘service user experiences’ and ‘team activities’, summarized it (by paraphrasing interviewees key points) using the framework function in NVivo 9 (QSR International) and used the resulting framework matrix to identify the service user experiences and team activities discussed by our interviewees.

Two team members (VW and LP) compared the service user experiences which we had identified from the local and national outcome frameworks, scoping reviews and interviews. We discussed, grouped and summarised these to develop a final list of six service user experiences associated with integrated care. We used the same approach to develop a list of 15 attributes and activities associated with integrated teamworking.

##### Phase 2: developing a logic model

The second phase of the project involved linking the service user experiences and team activities and developing a series of ‘logic models’ [23]. This type of model is widely used in program evaluations as a way of helping program developers to clarify their goals and planned activities and for visualizing the linkages between them. Since our overall aim was to develop a framework which would enable staff working in the neighbourhood teams to explicitly understand the links between service user experiences and their activities and working practices (and make any necessary changes), we judged that logic models could be a useful part of this framework. The logic models were also designed to act as a tentative roadmap for service providers and commissioners by showing how the Integrated Neighbourhood Teams were designed to work and where any adjustments needed to be made. Recognising that it was important to co-produce the logic models with staff who had a more detailed view of the Integrated Neighbourhood Teams, we held a 2 h workshop with local commissioning and operational managers and a service user involvement specialist. During the workshop we worked with these managers to group together the team activities which were most likely to influence each of the six service user experiences and to arrange them into a logical chain of events. We used the outputs from this meeting to produce draft versions of the models which were circulated to our project collaborators for further comments and adaptation.

##### Phase 3: devising tools for gathering experiences of integrated care

The third phase of the project involved devising tools for collecting and analysing service user experiences. Some of our project partners originally envisioned that survey tools could be used to collect service user experiences but after a number of group discussions about the difficulties of administering and collecting surveys from the frail older adults who were most likely to be receiving care via an Integrated Neighbourhood Team [26] and the limitations of survey data for understanding individual experiences, we decided to focus on capturing rich descriptions of people’s experiences in their own words using a qualitative approach [27].

We recognised that qualitative approaches have the potential to generate a vast amount of data which can be difficult and time-consuming to analyse. This had the potential to clash with our overall aim of developing a practical framework which could be used by staff with relatively little research experience. One team member (GF) therefore reviewed a range of academic and grey literature about the collection and analysis of qualitative data by lay researchers and service users [28–30] and identified a number of key principles. These included dividing the data collection and analysis process into a series of clear and well-defined tasks, developing a topic guide flexible enough to meet the needs of interviewees, using a simplified coding framework and developing creative ways of presenting findings that may help to overcome resistance from staff members. We drew on these principles and our own experience and knowledge of qualitative analysis techniques to devise a comprehensive series of data collection and analysis tasks, a procedure for working with interview data direct from audio recordings [31], a simple analysis codebook [32] and a procedure for comparing and summarizing service user experiences into a single narrative story. As with the previous phases, we worked closely with our local partners in developing these materials and approaches.

##### Phase 4: devising tools for developing service improvement plans

The final phase of the project focused on devising a procedure to enable the Integrated Neighbourhood Teams to reflect on and develop their teamworking practices by reflecting on the experiences of their service users. To develop the procedure we drew on two main resources. In line with our overall emphasis for the project, the first was developmental evaluation methodology [23], which focuses on the innovative use of formative feedback (and other resources) to improve and develop an ongoing intervention (such as integrated neighbourhood teams). The second was NHS service improvement resources [33]. As well as focusing on facilitating collaborative problem-solving, understanding and mapping current practices and planning activities to improve those practices, many of these have also been developed and tested in NHS contexts and were familiar to most of our partner organisations. In collaboration with our project partners, we used these sources to devise a structured protocol which would enable teams to reflect on the experiences of their service users, identify how their work impacts on those experiences and develop a clear service improvement plan to address any identified issues.

Between January and April 2014 the SUFFICE framework was implemented with three Integrated Neighbourhood Teams by our partner organisations. They took full responsibility for implementing the framework (including collecting and analysing the service user feedback data and arranging service improvement planning meetings). In the interests of data protection and brevity, we cannot present the results of the work conducted by our partner organisations or comment on the implementation of the SUFFICE framework.

In the following section we describe the contents of the SUFFICE framework in more detail.

## Results

### Suffice framework materials

In this section we describe the SUFFICE framework materials in the order in which they were developed. We begin by presenting the outputs of phases 1 and 2—our models of the expected linkages between integrated care experiences and practices. We then present the tools for gathering integrated care experiences (phase 3) before detailing the tools for developing service improvement plans (phase 4). In practice, the SUFFICE materials are designed to be used in a different order to that in which they were developed, and so we round the section off with a flow diagram showing how the SUFFICE framework is designed to be operationalised (Fig. 2).

#### Logic models: linking integrated care experiences and practices

From our literature review, interviews and review of previous outcome frameworks we identified six service user experiences which are expected to arise from an increase in integrated working. To make them readily accessible to a range of audiences, and in line with the influential work undertaken by National Voices to produce coordinated care narratives [25] and advice from our project partners, we expressed these as a series of ‘I’ statements. Four statements are related to a nominal service users’ journey, from assessment through to receipt of care from a new service, whilst the other two relate to care processes in general.

1. When my needs are being assessed and my package of care is being put together (or altered) I do not keep having to say the same thing to lots of different people.
2. When the care and support I need has been agreed, I receive it in an efficient and timely manner—things happen when they are supposed to.
3. When my needs change or things go wrong I know who to contact/who to go to/what to do—I am not bounced around the system.
4. When I need care and support from a new service (e.g. hospital), they already know what my needs are and who else is involved in providing me with care and support.
5. My package of care and support is focused on me and my needs—my opinion is listened to and respected.
6. I know about the range of formal and informal support that is available to me.

From our literature review and interviews we identified 15 markers of and activities associated with integrated teamworking.

1. Teams have networks across a number of agencies and work closely with those agencies.
2. Team members are able to work across geographical boundaries.
3. Team members understand one another’s background and culture.
4. Teams offer a full range of support. They are able to access and signpost to specialist and community support.
5. Teams have a shared identity in which all team members are engaged.
6. Teams clearly identify one case manager for each service user.
7. Teams identify who is best placed to do an assessment or care planning.
8. The team carries out one joint comprehensive assessment.
9. The team shares information with other agencies about individual service users.
10. Staff are able to blur boundaries and share work with each other.
11. Teams are comfortable working closely together.
12. Teams have regular meetings to discuss shared cases.
13. Teams respond quickly to service user need.
14. Team members are focused on their service user’s needs.
15. Teams are able to make efficient use of time.

**Fig. 1:**
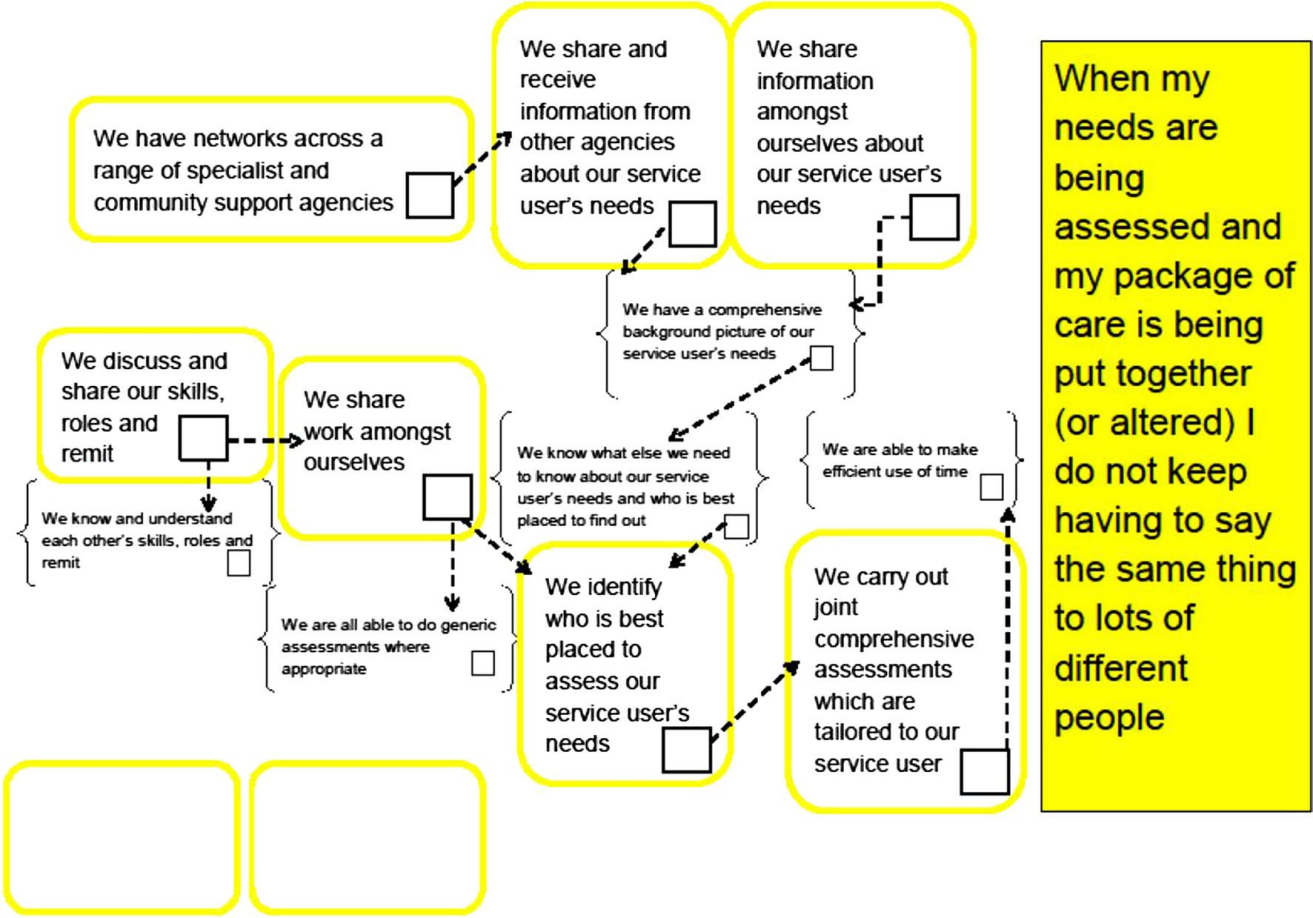
‘Assessment’ logic model. A model showing the expected logical progression and linkages between team activities (shown in *rounded boxes*), team-level outcomes (shown in *brackets*) and service user experience (shown in a *coloured box* on the *right* of the diagram)

During the workshop and subsequent discussions with local managers, these sets of expectations were adapted and linked together to produce a series of six ‘logic models’ (one for each expected service user experience). Each model includes team activities (shown in rounded boxes), team-level outcomes (shown in brackets) and the expected service user experience (shown in a coloured box on the right of the diagram). Dotted arrows link many of the team activities and outcomes to demonstrate the expected logical sequence of events. An example of a logic model can be seen in Fig. 1 below. All of the logic models can be found in Additional file 1: Appendix S1.

The main purpose of the logic models is to help facilitate structured reflection and the development of service improvement plans by team members. This led us to include several additional elements in each logic model. First, to facilitate structured reflection on the part of the teams, each activity and outcome is expressed from the perspective of the team (‘we’ statements). Second, each activity and outcome also includes a box for team members to indicate whether they feel that each of them occurs in their team. Finally, empty boxes are included so that they can also suggest other important activities not represented in the model.

#### Tools for gathering and analysing service user experiences

Since we were interested in gathering the experiences of service users in their (or their carers’) own words, the data collection tools are not driven by the six expected experiences represented in the logic models. Instead, we devised a semi-structured interview schedule divided into three sections, as shown in Table 2. The full interview schedule is available in Additional file 2: Appendix S2.

**Table 2:** overview of the service user experiences interview schedule with examples

Interview section	Description	Interview schedule example
Introduction/basic information	This section focuses on gathering basic details about the service user and their current situation. It is also designed to help the interviewer and interviewee develop a rapport	Please tell me a little about yourself/the person you care for Do you/they have any particular health conditions? How old are you/they?
Timeline	This section involves using a simple timeline to record significant events and experiences over the past 6–12 months. It is designed to be a visual tool to help focus the interview and identify key episodes to explore in more detail	Instruction to interviewer: Use the timeline tool to identify key events and to generate discussion about the interviewee’s experience of health and social care Question: Could you tell me about the care and support you/the person you are caring for has received over the last 6–12 months?
Key events/episodes	This section involves focusing in more detail on the key events experienced by the service user. It includes a series of prompts to help explore the different types of event that a service user might have experienced (assessment, receiving care and support, changing needs/crisis, accessing new services)	Instruction to interviewer: Using the timeline as a guide, focus on key events where things seemed to have gone well, along with those where things seemed to go wrong. Ask the interviewee to explore what happened and why they think things went well/badly. **Assessment prompts:** How much time did the person assessing/planning your care spend with you? Did it feel like enough time? How many people did you see during the assessment process? Who were they? What did they do/ask you? Where did you see them? Did you feel listened to?

The six expected service user experiences were used to drive the analysis plan, which is divided into three phases.

1. *Familiarisation and identifying relevant material*. This phase involves listening to an audio recording of each interview and noting the content using a timed grid. The aim of this phase is to identify the points at which various topics are discussed and start to identify material which relates to the six expected service user experiences.
2. *Coding and summarizing*. This phase involves listening in detail to key points of analytical interest (i.e. material which relates to the six service user experiences) and producing detailed summaries of this material guided by an analysis codebook. An extract from the codebook is shown in Table 3. The full analysis codebook can be found in Additional file 3: Appendix S3.
3. *Comparing and synthesizing*. This phase involves transferring the summaries produced for each interview into a simple table to enable the comparison of experiences between interviewees. The aim of this phase is to summarise the key points of similarity and difference in relation to each expected experience across several interviews. An example can be seen in Table 4.

**Table 3:** Extract from the service user experiences analysis codebook

Theme 1: When my needs are being assessed and my package of care is being put together (or altered) I do not have to keep saying the same thing to a lot of different people
This section focuses on assessments and how the plan of care was produced if at all. Focus on parts of the interview where the interviewee talks about: Someone talking to them about their needs Having their needs assessed How assessments were carried out How many/which people were involved in assessing their needs Having to repeat their needs to different people How their care was planned Who was involved in planning their care
Notes/summary
Interesting quotes

**Table 4:** Table for comparing and synthesizing service user experiences

	Interview 1	Interview 2	Summary of key points
Section 3 crisis	KW always knew which provider the care workers came from, but not who to contact in the event of a problem. There was no central number to call	At night, the only emergency number PB had was for a GP, but would have preferred a number for a nurse; She wonders whether other people, less willing to bother staff, might be less likely to get appropriate support	Both KW and PB experienced difficulties contacting staff in the event of an emergency

To enable these key points to be communicated to a range of audiences and used as the basis for developing service improvement plans, we devised a mechanism for constructing composite stories based on the experiences of several service users. Stories are increasingly being used as a way of communicating service user experiences and have been shown to be a powerful catalyst for service redesign and change by inspiring understanding and empathy, and encouraging service providers to listen, learn, and act upon what they are told [34, 35]. The mechanism we developed focuses on producing separate stories to illustrate each of the six service user experiences. Our materials include a simple template and style guide which make it clear that whilst the decision about how to weave together the key points rests with the analyst (which may include embellishing contextual details and circumstances), the focus of the story should remain on the key points which emerged from the analysis.

#### Tools for structured reflection and planning

We developed a structured protocol to guide teams through the process of developing a service improvement plan over the course of one or two meetings. The protocol includes four separate stages, drawing on the composite stories of service user experiences and the logic models, which culminate in an agreed service improvement action plan. The protocol also includes instructions about selecting a facilitator and scribe for the group discussion and someone to take responsibility for coordinating the implementation of the agreed service improvement plan. An overview of the four stages and example text from the protocol is shown in Table 5 below. The complete service improvement protocol can be seen in Additional file 4: Appendix S4.

**Table 5:** An overview of the service improvement protocol

Stage	Description	Improvement protocol example
Stage 1: storytelling and initial reactions	The team is told a composite story illustrating service user experiences in relation to one of the six expected experiences and has an opportunity to give some initial reactions. The aim is to provide everyone with an opportunity to air and ‘park’ any initial thoughts, reactions, questions or concerns so that they do not become a distraction during the following stages	After listening to the story, please invite team members to give one initial thought/impression and make a note of them here
Stage 2: identifying areas for improvement	Team members consider their activities and ways of working and how these may have influenced the story they have heard using the relevant logic model (the one which relates to the story they have been told) and a series of questions and prompts. The aim is to identify areas for improvement by identifying activities which the team tend not to engage in	Looking at the diagram, where did things go right in the story? What was working well for the service user? What activities did we seem to do? Where did things go wrong in the story? What wasn’t working well for the service user? What didn’t we seem to do? Use the boxes to indicate the things you did/didn’t do. Even if this isn’t clear from the story itself, use your experience to identify things you are likely to have done/not done
Stage 3: selecting an area for improvement	Team members select where to focus their service improvement efforts by discussing the results of the previous stage using a series of prompts. At the end of this stage, teams use the protocol to record their decisions about the activities that they have decided to focus on. If the process is to be carried over to a second meeting, teams also record the person who will lead/coordinate those efforts and the date by which they will have devised a concrete service improvement plan	Which activities are likely to have had the most influence on the service user story? What should we keep doing/do more of to deliver positive experiences for our service users? What do we need to start/fix to deliver better experiences for our service users?
Stage 4: developing a service improvement plan	Team members develop concrete plans for improving the selected activities using a series of prompts based on the ‘five whys’ principle [36]. This aims to uncover the root causes of teams’ ability or inability to carry out the activities which influence service user experiences which can act as a precursor to developing solutions. Guidance to help team members think creatively and positively about possible solutions is also included to counteract the tendency for teams to focus on what they are unable to do. At the end of this phase, teams complete a service improvement action plan which includes their planned activities, who is responsible for the activity, and the expected completion date	Why doesn’t this activity happen? Why don’t we do it? What can we do to address this? How can we check that this activity is important to our service users? How can we get feedback from our service users about our improvement plans? How can we monitor the success of our planned activities?

**Fig. 2:**
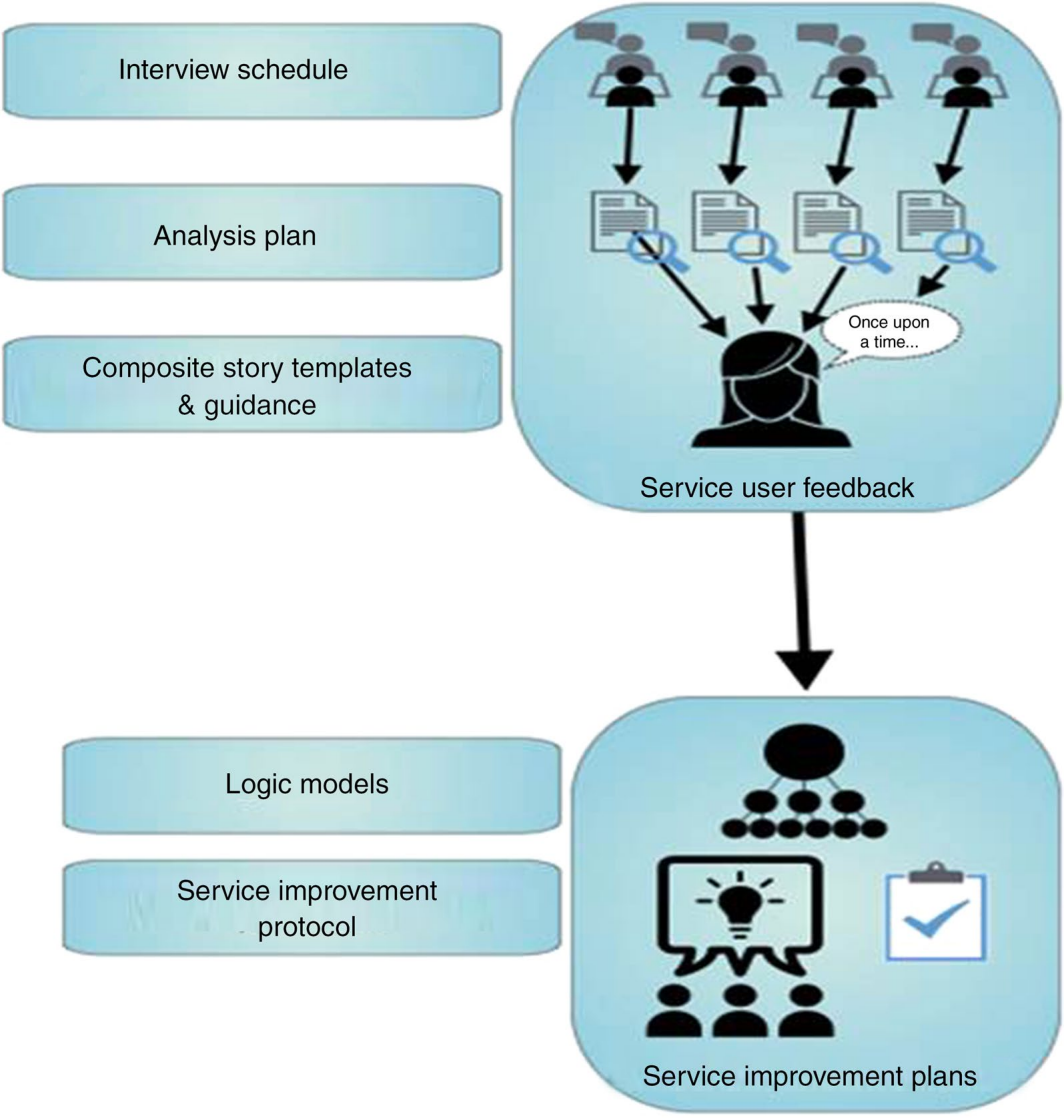
Using the SUFFICE materials in practice

The complete SUFFICE framework is shown in Fig. 2 below, which demonstrates how the materials we have described fit together in practice.

## Conclusions

In this paper, we have presented an innovative framework for gathering and using service user feedback to inform ongoing service improvement in integrated care.

The SUFFICE framework is important in two respects. First, gathering and evaluating service user experiences are neglected aspects of the integrated care landscape, with the focus tending to remain on idealised descriptions of integrated care from an organisational perspective. The SUFFICE framework aims to rebalance this by providing a mechanism for producing realistic composite stories which represent the voices and experiences of those receiving integrated care services. Second, enabling teams of staff to develop a shared sense of purpose focused on the needs of their service users is a crucial aspect of improving integrated working between health and social care staff. The SUFFICE framework provides a mechanism for teams to make plans for improving integrated working which have service user experiences at their heart.

## Strengths and limitations

- The main strengths of this study are its collaborative design, its focus on developing a practical tool with real-world application and its focus on service user experience.
- The main limitations of this study are its focus on one geographical area in the United Kingdom and limited data on how the framework was implemented over time.

## Additional files

10.1186/s13104-016-2230-0 SUFFICE logic models.
10.1186/s13104-016-2230-0 SUFFICE service user interview schedule.
10.1186/s13104-016-2230-0 SUFFICE analysis template.
10.1186/s13104-016-2230-0 SUFFICE service improvement protocol.

## Abbreviations

SUFFICE: Service User Feedback Framework for Improving integrated CarE
NHS: National Health Service
UK: United Kingdom
GP: general practitioner
