# Supplementary material for: Developing a framework for gathering and using service user experiences to improve integrated health and social care: the SUFFICE framework
Source: BMC Res Notes. 2016 Sep 8;9(1):437. doi: 10.1186/s13104-016-2230-0 (PMC5017127; doi:10.1186/s13104-016-2230-0)
Supplement: Supplementary file 1 — 10.1186/s13104-016-2230-0 SUFFICE logic models. [file 13104_2016_2230_MOESM1_ESM.pdf]

Appendix 1: SUFFICE logic models

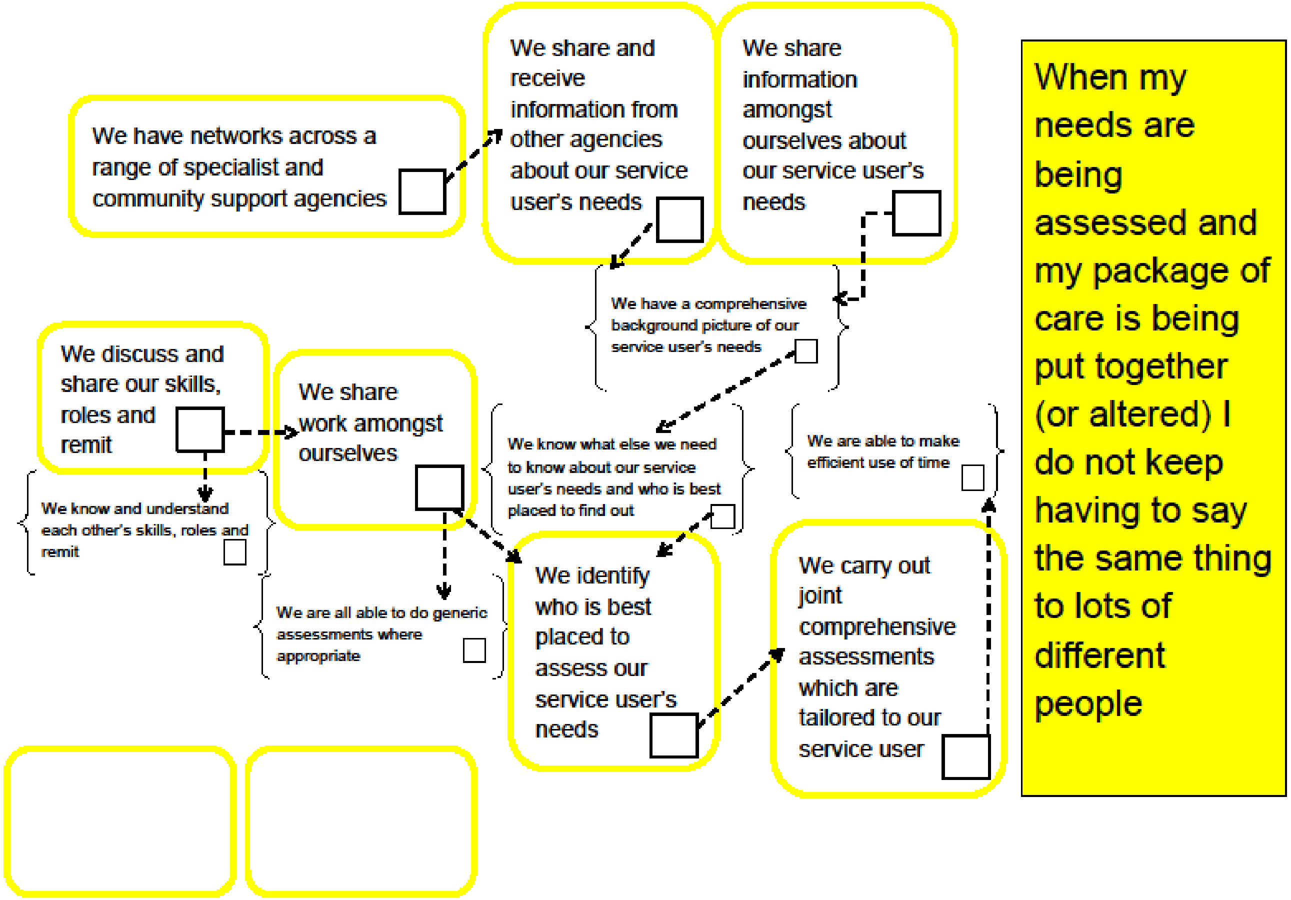

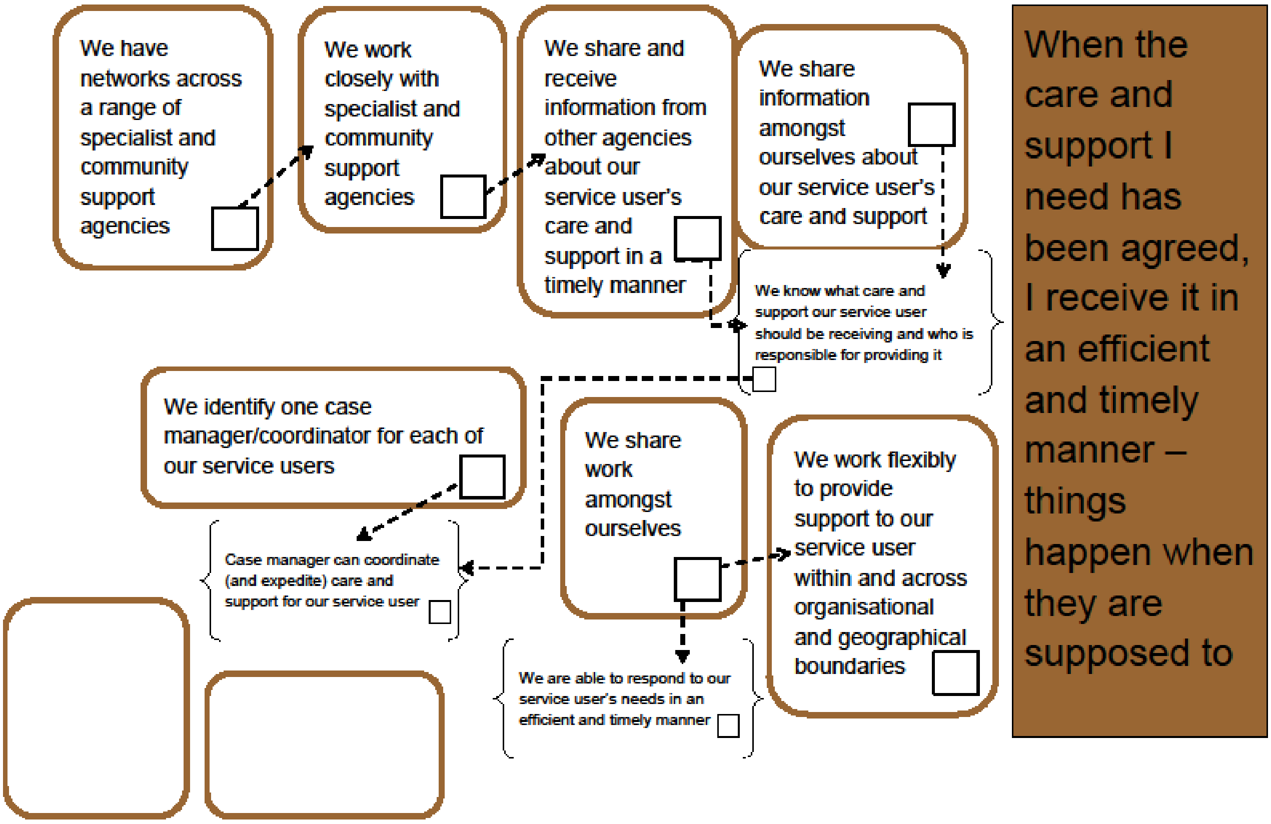
We have networks across a range of specialist and community support agencies
We work closely with specialist and community support agencies
We share and receive information from other agencies about our service user's care and support in a timely manner
We share information amongst ourselves about our service user's care and support
We know what care and support our service user should be receiving and who is responsible for providing it
We identify one case manager/coordinator for each of our service users
Case manager can coordinate (and expedite) care and support for our service user
We share work amongst ourselves
We work flexibly to provide support to our service user within and across organisational and geographical boundaries
We are able to respond to our service user's needs in an efficient and timely manner
When the care and support I need has been agreed, I receive it in an efficient and timely manner – things happen when they are supposed to

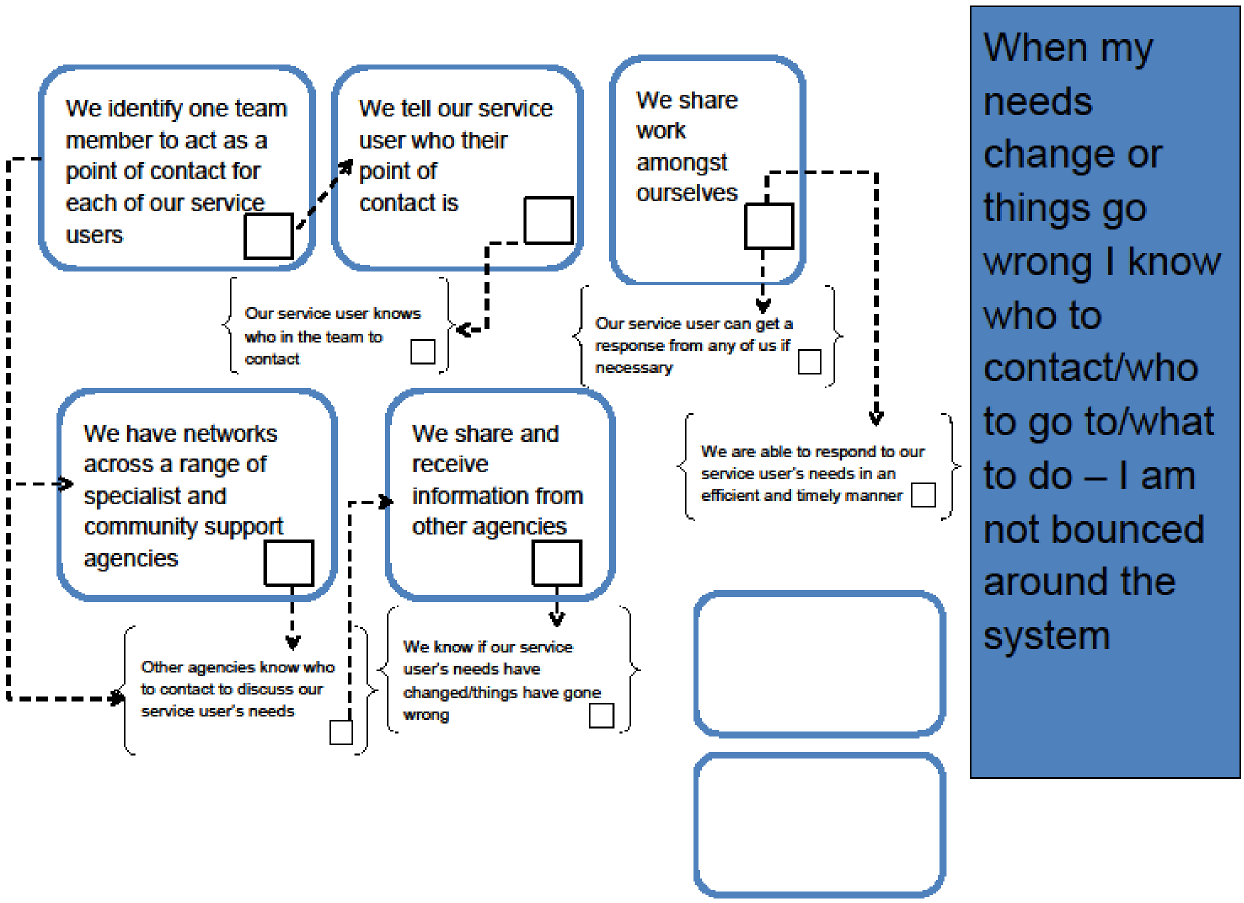
We identify one team member to act as a point of contact for each of our service users
We tell our service user who their point of contact is
We share work amongst ourselves
Our service user knows who in the team to contact
Our service user can get a response from any of us if necessary
We are able to respond to our service user's needs in an efficient and timely manner
We have networks across a range of specialist and community support agencies
We share and receive information from other agencies
Other agencies know who to contact to discuss our service user's needs
We know if our service user's needs have changed/things have gone wrong
When my needs change or things go wrong I know who to contact/who to go to/what to do – I am not bounced around the system

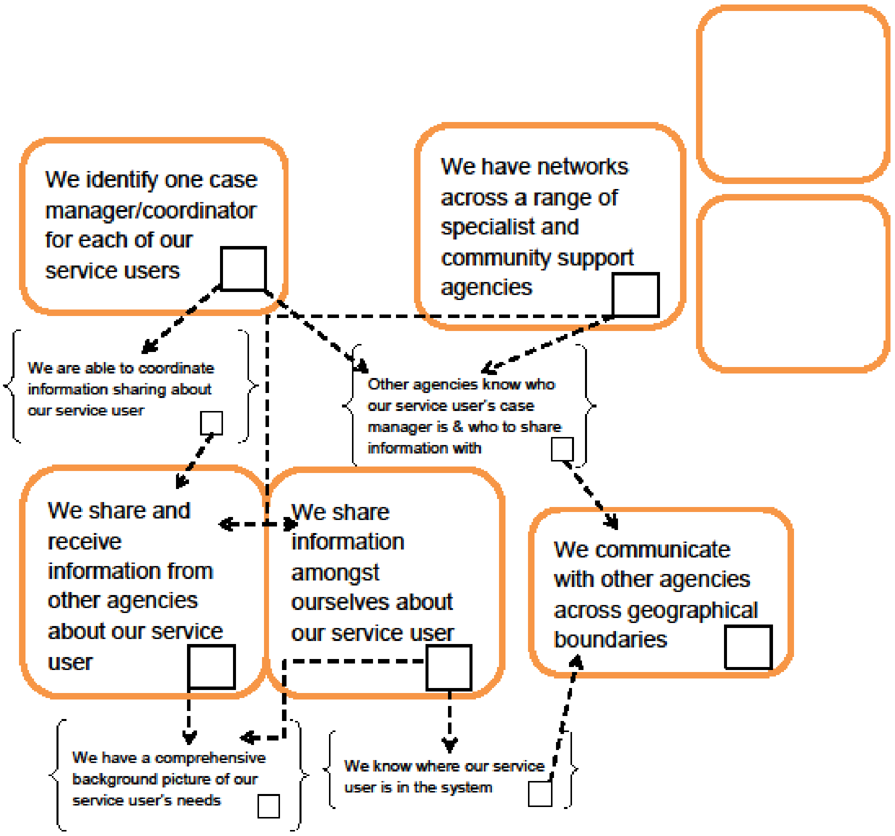

When I need care and support from a new service they already know what my needs are and who else is involved in providing me with care and support

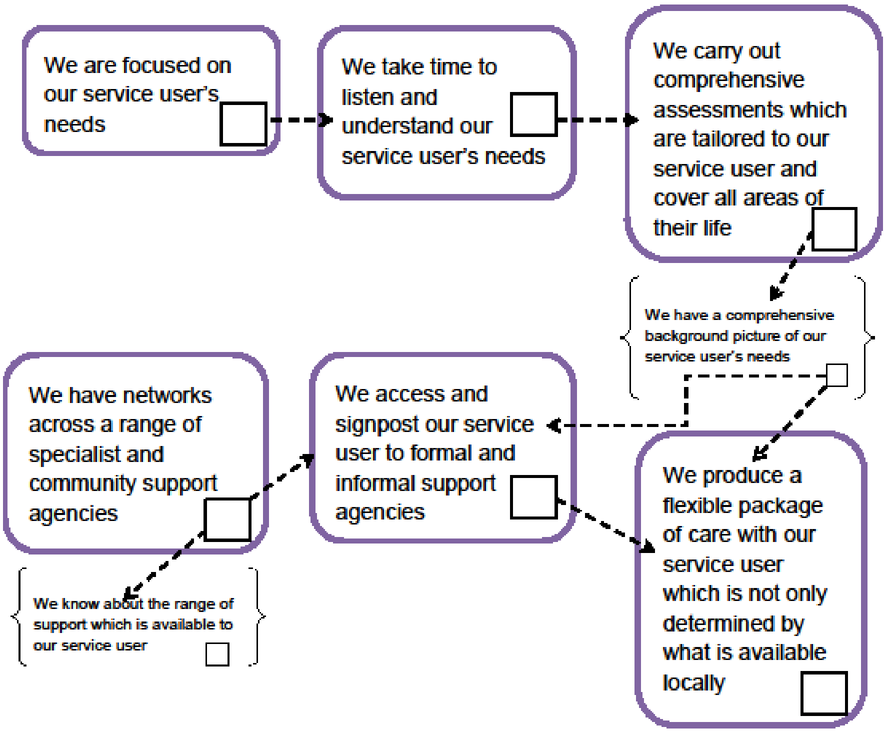

My package of care and support is focused on me and my needs – my opinion is listened to and respected

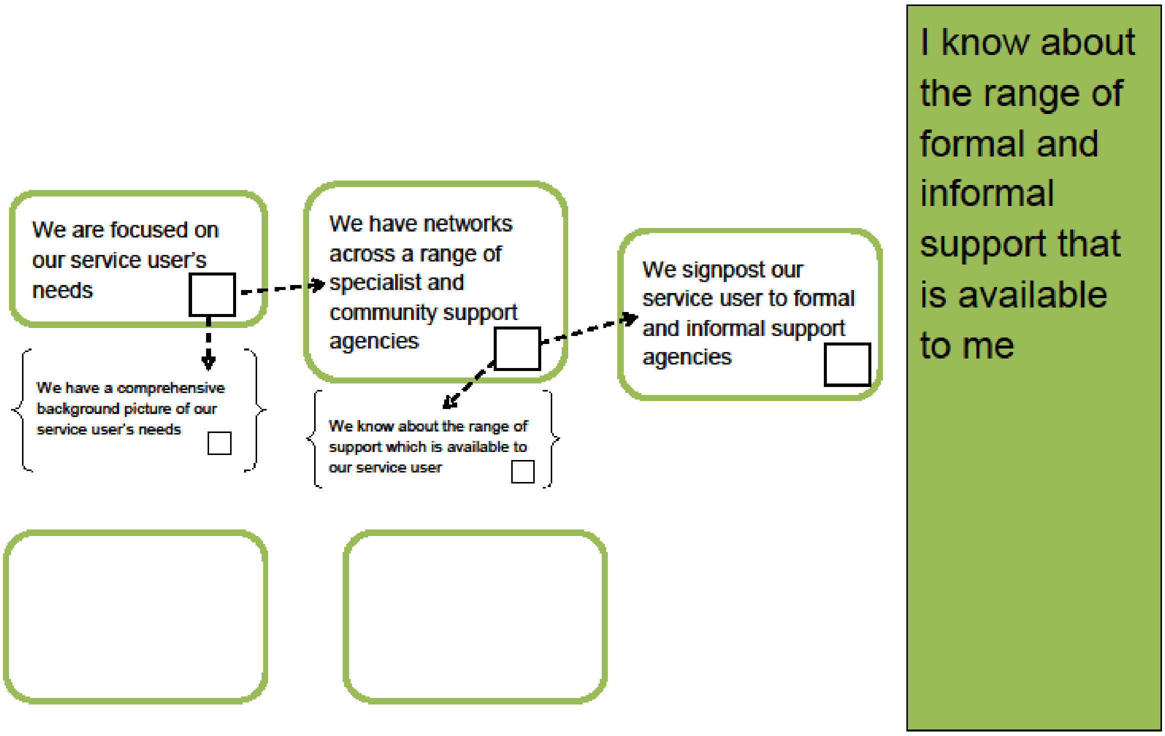
I know about the range of formal and informal support that is available to me
We are focused on our service user's needs
We have a comprehensive background picture of our service user's needs
We have networks across a range of specialist and community support agencies
We know about the range of support which is available to our service user
We signpost our service user to formal and informal support agencies
