# Supplementary material for: Developing a framework for gathering and using service user experiences to improve integrated health and social care: the SUFFICE framework
Source: BMC Res Notes. 2016 Sep 8;9(1):437. doi: 10.1186/s13104-016-2230-0 (PMC5017127; doi:10.1186/s13104-016-2230-0)
Supplement: Supplementary file 2 — 10.1186/s13104-016-2230-0 SUFFICE service user interview schedule. [file 13104_2016_2230_MOESM2_ESM.pdf]

Appendix 2: SUFFICE Service user interview schedule

Section 1: Introductory screening questions / basic information

Please tell me a little about yourself / the person you care for.

Do you / they have any particular health conditions? (Explore the health status of the interviewee / cared for person.)

How old are you / they?

Section 2: Timeline

Could you tell us about the care and support you/the person you are caring for has received over the last 6 to 12 months?

Use the timeline tool to identify key events and to generate discussion about the interviewee's experience of health and social care.

Visual tool: Timeline

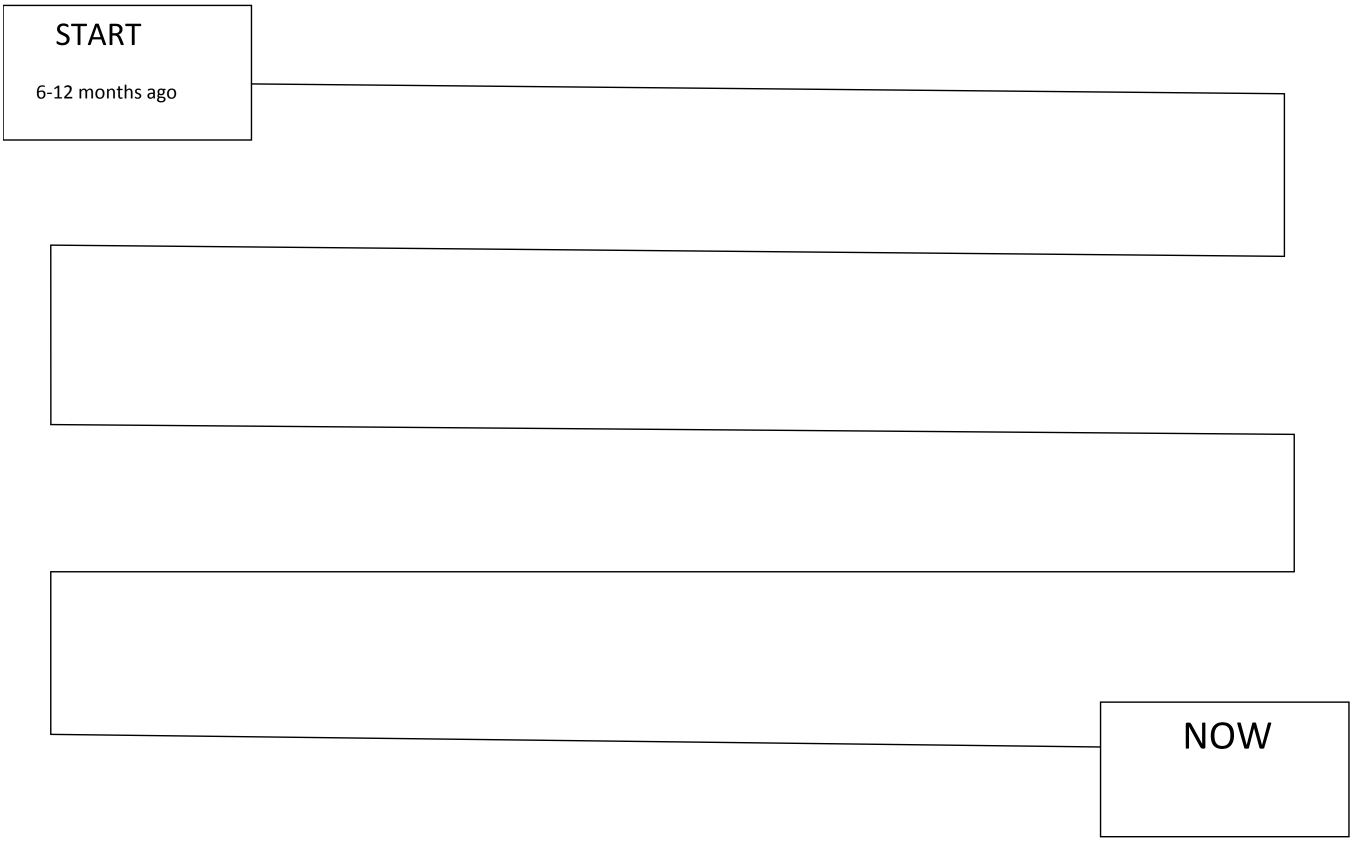

Section 3: Key events

Focus on key events where things seemed to have gone well, as well as on those where things seemed to go wrong. Ask the interviewee to explore what happened and why they think things went well/badly.

Ask the interviewee to help you identify what kind of issue each event related to: **Assessment of need, receiving care and support, changing needs, new services.** Use the prompts from the relevant sections (below) to help the discussion and explore each issue in sufficient detail.

Section 4: Prompts by type of event

Event	Prompts
**Assessment of need/care planning**	How much time did the person assessing/planning your care spend with you?<br>How many people did you see during the assessment process?<br>• Who were they?<br>• What did they do/ask you?<br>• Where did you see them?<br>Did you feel listened to?<br>Did you feel there was appropriate time for the assessment feel (e.g. feel rushed)?<br>Was the care package that was produced appropriate for your needs? Were you happy with it?
**Receiving care and support**	What kinds of care and support do you access?<br>Are you aware of what care and support is available to you locally?<br>Did you know how long it would take to receive the care and support you required?<br>Were you told what to expect in terms of timings and process?<br>Did you access all services you required and / or were promised?<br>Has the amount of time it has taken to get appropriate care and support changed since this early episode (i.e. is it quicker / slower)?<br>What did you feel about the service you received (i.e. un/happy with it?)?<br>In what way could things have been improved?
**Changing needs and crisis points**	Did you ask anyone for help?<br>Which people did you see/contact? How many?<br>How did you know who to contact?<br>Did/do you know who was providing your care?<br>Did/do you know what they could/would do for you?<br>Did you feel supported emotionally/practically? If so, who by?

**Accessing new services**	Did you have to repeat things when you accessed new services? If not, did people involved understand what your needs were?<br><br>Did people involved in your care share appropriate information about you? If so, do you know how this was done?<br><br>Did people involved in your new care know who else was involved in providing your care and support?<br><br>Did you always know what was happening in your care and support? If so, how was this communicated to you?<br><br>Did you access all services you required and / or were promised?
