# Supplementary material for: Developing a framework for gathering and using service user experiences to improve integrated health and social care: the SUFFICE framework
Source: BMC Res Notes. 2016 Sep 8;9(1):437. doi: 10.1186/s13104-016-2230-0 (PMC5017127; doi:10.1186/s13104-016-2230-0)
Supplement: Supplementary file 3 — 10.1186/s13104-016-2230-0 SUFFICE analysis template. [file 13104_2016_2230_MOESM3_ESM.pdf]

Appendix 3: SUFFICE Analysis template

Part 1: Evaluator details
Participant ID
Evaluator No
Interview completed date
Analysis completed date
Interview location details
Feedback date
Reflexive diary/thoughts. Include any assumptions, thoughts and experiences which might influence how you hear and interpret the interview data.
Part 2: Service user demographics/background information/history
A full description of the service user and their circumstances. Also include relevant details about the interviewee (if they are not the service user).
Part 3: Themes from interviews
**THEME 1: When my needs are being assessed and my package of care is being put together (or altered) I do not have to keep saying the same thing to a lot of different people**<br>Notes: This section focuses on assessments and how the plan of care was produced if at all. Focus on parts of the interview where the interviewee talks about:<br>• Someone talking to them about their needs<br>• Having their needs assessed<br>• How assessments were carried out<br>• How many/which people were involved in assessing their needs<br>• Having to repeat their needs to different people<br>• How their care was planned<br>• Who was involved in planning their care
Interesting quotes:

**THEME 2: When the care and support I need has been agreed, I receive it in an efficient and timely manner-things happen when they are supposed to.**<br>Notes: This section focuses on the service user's experience of receiving care. Focus particularly on parts of the interview where the interviewee talks about:<br>• How quickly things were put into place for them<br>• How long they had to wait for care and support<br>• How quickly they expected things to be put into place and whether these expectations were met<br>• How satisfied they were with the care and support they received<br>• How health and social care staff communicated with them about the care and support they would receive
Interesting quotes:
**THEME 3: When my needs change or things go wrong I know who to contact/who to go to/what to do-I am not bounced around the system**<br>Notes: This section covers changing needs (which could include getting better) and crises. Focus particularly on parts of the interview where the interviewee talks about<br>• Experiences of asking people for help<br>• Knowing who to contact and how to contact them<br>• Experiences of trying to contact people when their needs changed<br>• Their ability to access the right care and support when their needs changed/in a crisis<br>• How 'joined up' the response to their changing needs/crisis appeared to be
Interesting quotes:

**THEME 4: When I need care and support from a new service (e.g. hospital) they already know what my needs are and who else is involved in providing me with care and support.**<br>Notes: This section focuses on whether the services accessed by the service user were joined up. Focus on parts of the interview where the interviewee talks about:<br>• Communication and information sharing between the professionals involved in their care<br>• Communication and information sharing across services<br>• Experiences of accessing new services<br>• How much staff knew about them/their needs
Interesting quotes:
**THEME 5: I know about the range of formal and informal support that is available to me**<br>Notes: This section focuses on the range of care and support that the service user knows about and accesses. Focus particularly on parts of the interview where the interviewee talks about:<br>• The kinds of support they know about and access<br>• Support offered by friends, family and neighbours<br>• Their involvement in their local community or voluntary sector schemes
Interesting quotes:

THEME 6. My package of care and support is focused on me and my needs - my opinion is listened to and respected

Notes: This section focuses on whether service users feel empowered to look after themselves and whether they feel that they are involved in decisions about their care. Focus particularly on those parts of the interview where the interviewee talks about:

- Making decisions jointly with health and social care staff
- Feeling listened to
- Feeling supported (emotionally, practically, spiritually)
- Their ability to say what they want and/or need
- Whether they receive the care and support that they want and/or need

Interesting quotes:

PART 4: Any extra information not covered by previous sections
