# Supplementary material for: Developing a framework for gathering and using service user experiences to improve integrated health and social care: the SUFFICE framework
Source: BMC Res Notes. 2016 Sep 8;9(1):437. doi: 10.1186/s13104-016-2230-0 (PMC5017127; doi:10.1186/s13104-016-2230-0)
Supplement: Supplementary file 4 — 10.1186/s13104-016-2230-0 SUFFICE service improvement protocol. [file 13104_2016_2230_MOESM4_ESM.pdf]

Appendix 4: SUFFICE Service improvement protocol

Team details
Neighbourhood Team:
Meeting date:
Names & roles of those present:
Name of storyteller:
Facilitator/lead[1]:
Scribe[2]:
Improvement lead[3]:
Story topic:
Part 1: Initial reactions (5 minutes)
After listening to the story, please invite team members to give one initial thought/impression and note them here
Part 2: Identifying the issues (10 minutes)
Please look at the diagram you have been given and use this to help you identify how activities you carry out as a team may have influenced the service user story[4] and where there is room for improvement. Ask any of the following questions to guide your discussion:<br>• Looking at the diagram, where did things go right in the story? What was working well for the service user? What activities did we do?<br>• Where did things go wrong in the story? What wasn't working well for the service user? What didn't we do?<br>You might like to place a tick or a cross in each of the boxes in the diagram to show the things you did/didn't do[5]<br>• Are there other activities we carry out as a team that may have influenced the service user story? Are there other places where things might have gone right/wrong that are not on the diagram?<br>If so, please write these in the empty boxes<br>Please write notes of your discussion here and write on the diagram you have been given.

[1] At the beginning of the meeting, please decide who will be responsible for facilitating the discussion and asking the questions in the following sections
[2] At the beginning of the meeting, please identify someone who will keep notes during the discussion
[3] Towards the end of the meeting, please identify someone who will be responsible for coordinating the future planning and implementation of activities to address the issue which you have identified
[4] For this section try to stay focused on the service user and think about things from their perspective
[5] Even if it is not clear from the story what activities you did/didn't do, use your experience to identify things you are likely to have done/not done

**PART 3: Selecting the issues to focus on (10 minutes)**
Using the results of your previous discussion, identify which issue(s) your team needs to do something about. Use the following questions to help you make your decision<br>• Which activities are likely to have had the most influence on the service user story[6]?<br>• What should we keep doing/do more of to deliver positive experiences for our service users?<br>• What do we need to start/fix to deliver better experiences for our service users?<br>Please write notes of your discussion here
**Our decisions**
As a team we have decided to do something about:
The person who will lead/coordinate our efforts to do something is:
The date by which we will have made concrete plans is:

[6] These can be elements where you have said ‘no, we don’t do that’ or ‘yes, we do that’. The important thing is that the element is a crucial part of providing service users with a positive experience.

PART 4: Developing concrete plans (after the session)
Now that you have identified an issue that you want to focus on as a team, you will need to identify things which you can do to address it[7]. Use the questions below to help you come up with some clear plans[8]. The questions you ask will depend on the type of issue you identified during the feedback session.<br>If you decided to focus on doing more of an activity which is important for your service users:<br>• How do we know this happens?<br>• Why does it happen? Why do we do it?[9]<br>• How can we make sure it continues to happen for other service users?<br>If you decided to focus on starting/fixing an activity which is important for your service users ask:<br>• Why doesn't this happen? Why don't we do it? (see footnote 9)<br>• What can we do to address this?<br>• How can we do it for other service users?<br>Also consider:<br>○ How can we check that this activity is important to our service users?<br>○ How can we get feedback from our service users about our improvement plans?<br>○ How can we monitor the success of our planned activities?

[7] Try to think creatively and focus on those things that you can do to address the issue, even if this seems very small. It is easy to get bogged down in finding reasons why you can't do anything.

[8] Actions can include using the results of the feedback to communicate/put your case more clearly to senior managers for changing things elsewhere in the system

[9] Keep asking the question 'why' as you discuss this question. Very often the first reason that is given doesn't go quite far enough in identifying the real reason that things do or don't happen. For an example see: http://www.institute.nhs.uk/quality_and_service_improvement_tools/quality_and_service_improvement_tools/identifying_problems_-_root_cause_analysis_using5_whys.html

Our action plan		
**Planned activity**	**Person responsible**	**Expected completion date**
